# A Sternoclavicular Joint-Specific Plate for the Displaced Medial-End Clavicle Fracture

**DOI:** 10.3389/fsurg.2022.875475

**Published:** 2022-04-29

**Authors:** Yunli Zhu, Fucun Liu, Lie Lin, Chuanyi Zhang, Bin Wang, Shouli Wang

**Affiliations:** ^1^Department of Orthopedics, Changzheng Hospital, Naval Medical University, Shanghai, China; ^2^Department of Orthopedics, Taizhou Hospital of Zhejiang Province Affiliated to Wenzhou Medical University, Linhai, China

**Keywords:** medial-end, clavicle fractures, specific plate, surgery, trauma

## Abstract

**Objectives:**

This study aimed to introduce a sternoclavicular joint (SCJ)-specific plate for the treatment of medial-end clavicle fracture and evaluate the clinical and radiological results of this method.

**Methods:**

From January 2006 to December 2020, 31 patients with displaced medial-end clavicle fractures were included in this study, with 8 patients with accompanying SCJ dislocation. Abduction and forward elevation of the shoulder, the Visual Analogue Scale (VAS), and the American Shoulder and Elbow Surgeons Score (ASES) were used for evaluation before index surgery and at the latest follow-up.

**Results:**

After an average of 98.5 (range, 13 to 171) months, the mean VAS significantly decreased from 6.8 ± 1.0 preoperatively to 0.9 ± 0.8 at the latest follow-up (*P* < 0.001). The mean ASES score significantly increased from 34.3 ± 7.8 preoperatively to 90.2 ± 4.9 at the latest follow-up (*P* < 0.001). The mean abduction of the shoulder significantly increased from 72.1 ± 6.6 preoperatively to 169.5 ± 8.5 at the latest follow-up (*P* < 0.001). The mean forward elevation of the shoulder significantly increased from 97.1 ± 11.0 preoperatively to 163.1 ± 11.5 at the latest follow-up (*P* < 0.001). The union of all fractures was achieved, and all implants were removed. No loose or breakage of implants was observed. No vascular or nerve damage occurred during the operation.

**Conclusions:**

This SCJ-specific plate provided excellent long-term results for the treatment of displaced medial-end clavicle fractures and was an alternative implant for medial-end clavicle fractures with or without small or comminuted medial fragments, especially those associated with SCJ dislocation.

## Introduction

Medial clavicle fractures are rare injuries and are usually caused by high-energy trauma, accounting for 2.8–10.6% of clavicle fractures (1–5). Traditionally, medial clavicle fractures are treated non-operatively, even though they are displaced substantially, worrying about catastrophic intraoperative complications because the vital structures are near the medial clavicle (6). However, non-union was reported in 14 to 20% of displaced medial clavicles treated with non-operative therapy (3, 7), and non-union may cause severe pain and impair shoulder function (8). For those patients, surgery might be necessary. Recently, surgery for displaced medial clavicle fractures has shown excellent results with few complications (9–14), but surgical techniques for fractures with small medial or comminuted fragments are still challenging.

In this study, we reported our results using a sternoclavicularjoint (SCJ)-specific plate (Canwell, Jinhua, Zhejiang, China) to treat displaced medial-end clavicle fractures. We aim to offer our experience as a reasonable alternative implant for the treatment of medial-end clavicle fractures with or without SCJ dislocation, especially those with small or comminuted medial fragments.

## Methods and Materials

### Study Population

This study was approved by the ethical committee of our hospital, and a signed informed consent form was obtained from all patients included in the study. The clinical and radiological data of patients with medial-end clavicle fractures treated with this specific plate were retrospectively reviewed between January 2006 and December 2020.

The indication for surgery was a displaced medial clavicle fracture, and all patients were treated with this specific plate between 2006 and 2020. The inclusion criterion for this study was traumatic medial-end clavicle fractures with or without SCJ dislocation. The exclusion criteria for this study were as follows: (1) a comorbidity of ipsilateral shoulder girdle injury, (2) previous ipsilateral clavicle fracture or dislocation of the SCJ, and (3) patients who were out of follow-up.

#### The SCJ-Specific Plate

**Figure 1:**
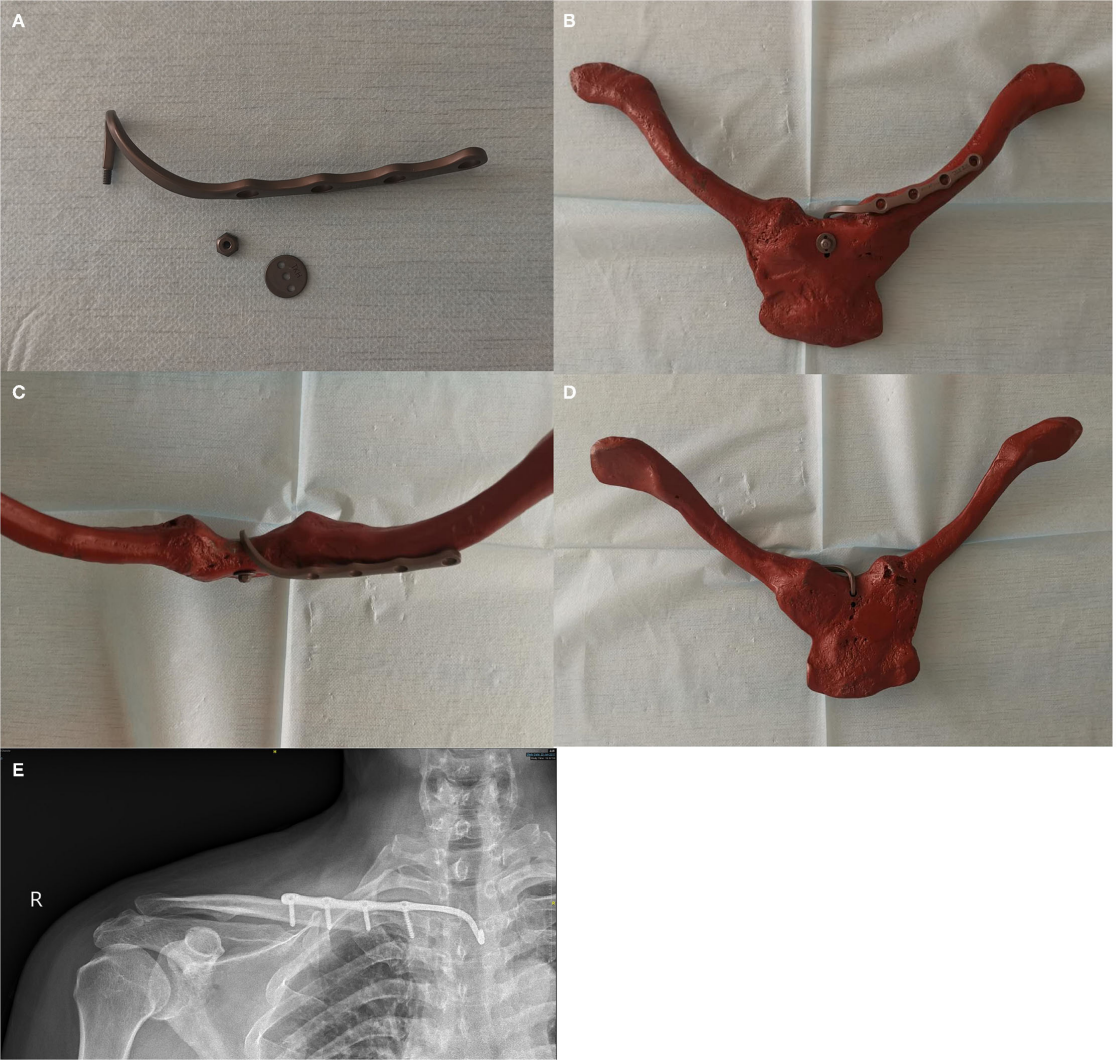
Sternoclavicular-joint specific plate **(A)** and its appearance fixed in a specimen from different directions [**(B)** anterior view; **(C)** superior view; **(D)** posterior view] and in postoperative X-ray **(E)**.

**Figure 2:**
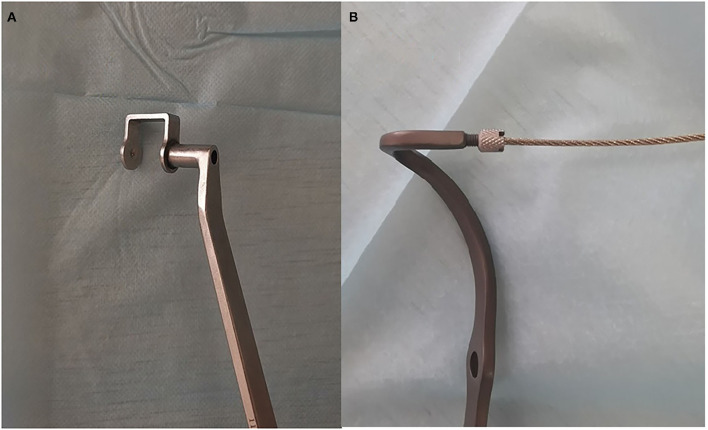
Special instruments used for surgery **(A)** Special drill jig used for the sternum, which is important to prevent the bit from damaging retrosternal tissues. **(B)** The guide cable is fixed with the hook of the plate and can guide the hook of the plate through the hole of the sternum conveniently from the posterior sternum.

An SCJ-specific plate consists of a plate and a hook that could be obtained in China. The plate is fixed to the anterior part of the distal fracture with three to four bicortical screws, while a hook is inserted into the hole created in the upper part of the manubrium from its posterior aspect with the help of the guide cable. Nuts and washers are fixed with the end of the hook if posterior or multidirectional instability occurs (Figure 1). This SCJ-specific plate was implanted with special instruments that were used to prevent damaging retrosternal vital structures and made this surgery more convenient (Figure 2).

#### Surgical Procedure

Surgeries were performed under general anesthesia with patients in the supine position. An inverted L incision along the proximal clavicle and sternal manubrium was made. The proximal clavicle, including the fractures, sternal manubrium, and ipsilateral SCJ, was exposed. After the fractures were temporarily reduced, a hole was made in the middle of the sternal manubrium, approximately 12 mm distal to the upper margin of the sternal manubrium, with the help of a special drill jig, which could prevent the drill from damaging the important retrosternal tissues. The hook of the specific plate was inserted into the hole of the sternal manubrium from back to front with the help of a guide wire. Three to four bicortical screws were used to fix distal fractures. Ligaments of the SCJ were repaired in situations of SCJ dislocation. Washers and nuts were used if posterior dislocation or multidirectional stability existed in situations of accompanying SCJ dislocation.

#### Postoperative Management

All patients were encouraged to perform passive exercises of the shoulder for the first month postoperatively, and active exercises were encouraged for the next month postoperatively. Basic daily tasks were resumed 2 months postoperatively. Implants were removed with evidence of the union of fractures.

#### Outcome Assessment

Postoperative follow-up was performed at 1, 3, 6 months, 1 year, and the latest follow-up. Computed tomography scans of the clavicle were performed before the index surgery and removal of the implant. X-rays were collected to assess bone healing at follow-up.

Abduction and forward elevation of the shoulder, the Visual Analogue Scale (VAS) (15), and the American Shoulder and Elbow Surgeons Score (ASES) (16) were used to evaluate clinical outcomes during follow-up.

### Statistical Methods

The statistical data analysis was performed with SPSS Statistics for Windows, version 22.0 (IBM Corp., Armonk, NY, USA). The measurement data were expressed as the mean ± SD and ranges. The clinical parameters were compared with independent *t*-tests. *P*-values <0.05 were considered significant.

## Results

**Figure 3:**
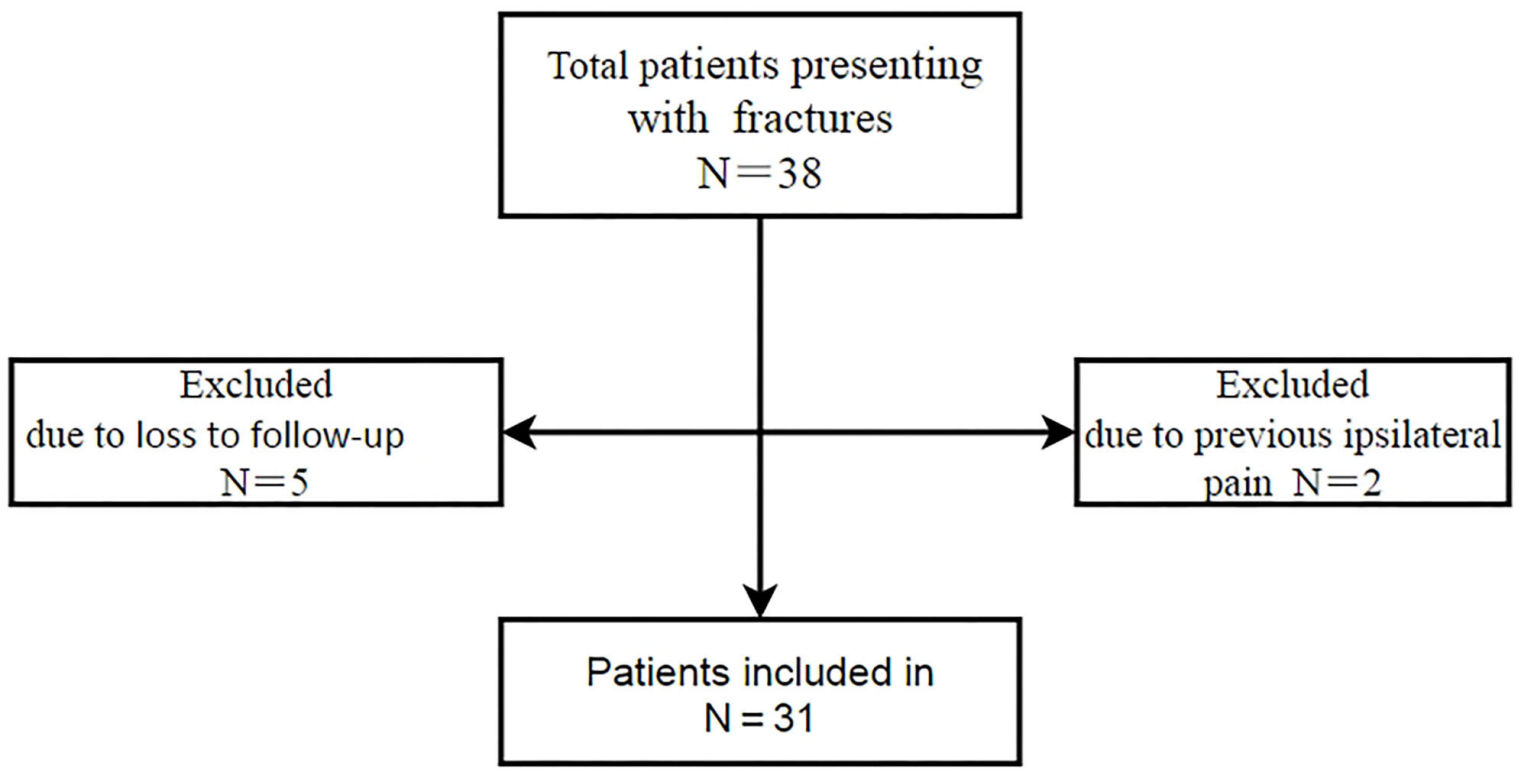
Flow chart of included patients treated with the specific plate.

From January 2006 to December 2020, 38 patients with displaced medial-end clavicle fractures were consecutively treated with this specific plate, with 2 patients excluded for their previous ipsilateral shoulder pain and 5 patients were lost to follow-up. Thirty-one patients were included in this study (Figure 3). Patient demographic data are shown in Table 1. The median age at injury was 47.5 (range, 29–72) years, with 19 male patients and 12 female patients. There were 15 left-side and 16 right-side fractures. The mean follow-up was 98.5 (range, 13–171) months. The mechanisms of injury included 19 traffic accidents, 8 falls from a height, 3 e-bike injuries, and 1 direct blow. Twenty-four cases were type 1B1 and 7 cases were 1B2 according to Edinburgh classification.

**Table 1:** Demographic data for the 31 patients with displaced medial-end clavicle fracture.

**Characteristic**	**Frequency count (%) or average (range)**
Sex	
Female	12 (38.7)
Male	19 (61.3)
Age (year)	47.5 (29–72)
Direction	
Left	15 (48.4)
Right	16 (51.6)
Follow-up (month)	98.5 (13-171)
Injury mechanism	
Traffic accident	19 (61.3)
Falling from a height	8 (25.8)
E-bike injuries	3(9.7)
Direct blow	1 (3)
Edinburgh classification	
Type 1B1	24 (77.4)
Type 1B2	7 (22.6)

Associated injuries included rib fractures, SCJ dislocations, vertebral fractures, facial fractures, hip fractures, and SCJ dislocation.

The mean VAS score significantly decreased from 6.8 ± 1.0 preoperatively to 0.9 ± 0.8 at the latest follow-up (*P* < 0.001). The mean ASES significantly increased from 34.3 ± 7.8 preoperatively to 90.2 ± 4.9 at the latest follow-up (*P* < 0.001). The mean value for the abduction of the shoulder significantly increased from 72.1 ± 6.6 preoperatively to 169.5 ± 8.5 at the latest follow-up (*P* < 0.001). The mean forward elevation of the shoulder significantly increased from 97.1 ± 11.0 preoperatively to 163.1 ± 11.5 at the latest follow-up (*P* < 0.001) (Table 2).

**Table 2:** Clinical outcomes at the two time points during follow-up.

	**VAS**	**ASES**	**Abduction (degrees)**	**Forward elevation (degrees)**
Before index surgery	6.8 ± 1.0	34.3 ± 7.8	72.1 ± 6.6	97.1 ± 11.0
The latest follow-up	0.9 ± 0.8	90.2 ± 4.9	169.5 ± 8.5	163.1 ± 11.5
*P*-value	0.000	0.000	0.000	0.000

*VAS, the Visual Analogue Scale; ASES, the American Shoulder and Elbow Surgeons Score*.

### Radiologic Results

The union of all fractures was achieved on x-ray and the mean time of bone union was 5.3 ± 1.0 months. All patients had their implants removed after a mean time of 10.7 months (range 8–14) postoperatively. No loose implants or breakage of implants was observed.

### Complications

Enlargement of the hole in the sternum was widely observed during the removal of the implant. No vascular or nerve damage occurred during the operation, and no infection occurred.

## Discussion

This study reports excellent long-term results of displaced medial-end clavicle fractures treated with this specific plate. The average follow-up of this study was 98.5 months and to the best of our knowledge, was the longest. A medial fracture is defined as a fracture located within one-fifth of the clavicle bone lying medial to a vertical line drawn upward from the center of the first rib according to the Edinburgh Classification (3). Non-operative therapy was the gold standard for a medial clavicle fracture even when fractures were displaced. However, Throckmorton et al. reported 28% moderate to severe pain in 44 medial clavicle fractures treated with non-operative therapy (2). Thus, surgery for these displaced medial clavicle fractures may be necessary. The current trend is shifting from conservative therapy to surgery. However, there is no consensus on the indication for surgery, and there is no specific implant for the fixation of medial-end fractures.

For medial clavicle fractures, several fixation methods of implants were reported. First, if the medial fracture could provide enough room for fixation of two or more screws, locking plates or non-locking plates fixing the medial and lateral fragments were the most common methods and achieved excellent results (1, 9–11, 13, 14, 17–20). Medial screws are often unicortical to avoid damage to nearby vital structures. Second, if the medial fracture could not provide enough room for the fixation of at least two screws, or medial fractures were comminuted, K-wires were reported to be used with low strength which would delay early rehabilitation and with the risk of migration (21). Also, plate bridging of the SCJ could resolve this problem (12, 21). In this situation, the SCJ was also fixed rigidly, and the range of movement of the SCJ would be influenced, which might lead to joint stiffness. Moreover, for bridging one SCJ, the strength of unicortical screws for the sternum was not so strong that early rehabilitation would be prolonged and the high torsion forces during movement might lead to loose screws (12). Another method reported in the case of a comminuted medial-end clavicle fracture by Li et al. was a plate that bridges the two SCJs (22). Comparing with the bridging of one SCJ, the method of bridging two SCJs was stronger and rigid, but needed more exposure and making the two SCJs rigidly fixed, which may lead to the stiffness of the two SCJs. Other methods as only screw fixation and transosseous sutures were relative, not strong (14, 17). Third, for special medial clavicle fractures as physeal-type injuries, transosseous sutures were also reported (14), but the sutures are not very strong.

The specific plate in this study was similar to the plate bridging SCJ reported by Zheng et al. (12), while screws fixed with the sternum were substituted by the hook, and were more suitable if the medial fracture part could not provide enough room for fixation of at least two screws or the fracture was comminuted. Moreover, this implant was suitable for physeal-type injuries, as no screws were needed to fix the epiphyseal plate, while the transosseous suture technique was performed by Sidhu et al. which might not be as strong (14). Additionally, this plate was suitable if fractures were accompanied by SCJ dislocation, as 8 patients' medial fractures with dislocation in this study showed bone union and excellent clinical results.

At the same time, the fixation of this implant maintained the micromotion of the SCJ, which could not be achieved by plates bridging one or two SCJs (12, 22).

Concerns about injury to vital structures around the medial clavicle prevented surgery for displaced medial fractures. However, there was no report of catastrophic intraoperative complications among patients (23), and so did this study. Some measures were taken about this specific plate for the treatment of SCJ dislocation. First, the special drill jig could prevent the bit from damaging retrosternal structures when a hole in the sternum was made. Second, small space of approximately 1.5 cm × 1.5 cm was enough for the manipulation of the retrosternum. Third, the placement of the hook from the retrosternum was guided with a guide cable.

Limitations of this study are that the sample size was small and no control group was used. Micromotion of hook in the hole of sternum produced windshield wiper effect to enlarge of the hole and the large hole may lead to failure of the fixation, so implants needed to be removed, which required another surgery.

## Conclusion

This SCJ-specific plate provided excellent long-term results for the treatment of displaced medial-end clavicle fractures and was an alternative implant for medial-end clavicle fractures with or without small or comminuted medial fragments, especially those associated with SCJ dislocation.

## Data Availability Statement

The original contributions presented in the study are included in the article/supplementary material, further inquiries can be directed to the corresponding author/s.

## Ethics Statement

The studies involving human participants were reviewed and approved by the Institutional Medical Ethics Review Board of WenZhou Medical College Affiliated Taizhou Hospital: Taizhou Hospital of Zhejiang Province. The patients/participants provided their written informed consent to participate in this study. Written informed consent was obtained from the individual(s) for the publication of any potentially identifiable images or data included in this article.

## Author Contributions

YZ and FL: methodology and writing—original draft. LL: resources and validation. CZ: data curation and formal analysis. BW: resources. SW: supervision and writing—review and editing. All authors contributed to the article and approved the submitted version.

## Funding

This study was supported by the Projects of Medical and Health Technology in Zhejiang Province in China (Project Number: 2022KY1379). The funder belongs to a government organization and provided financial support.

## Conflict of Interest

The authors declare that the research was conducted in the absence of any commercial or financial relationships that could be construed as a potential conflict of interest.

## Publisher's Note

All claims expressed in this article are solely those of the authors and do not necessarily represent those of their affiliated organizations, or those of the publisher, the editors and the reviewers. Any product that may be evaluated in this article, or claim that may be made by its manufacturer, is not guaranteed or endorsed by the publisher.

## Abbreviations

SCJ: sternoclavicular joint
VAS: Visual Analogue Scale
ASES: American Shoulder and Elbow Surgeons Score.
